# Evolution of Next-Generation Multiplex Lateral Flow Immunoassays: From Engineered Nanomaterials to AI-Driven Detection

**DOI:** 10.3390/bios16050269

**Published:** 2026-05-07

**Authors:** Tan-Thanh Huynh, Duc-Thang Vo, Trong-Nghia Le

**Affiliations:** 1 School of Chemical Engineering, Tra Vinh University, Vinh Long 85000, Vietnam; htthanh@tvu.edu.vn; 2 International Advanced Technology Program, College of Engineering, National Taiwan University of Science and Technology, Taipei 106, Taiwan; dtvo@mail.ntust.edu.tw; 3 Department of Chemistry, National Taiwan Normal University, Taipei 116, Taiwan

**Keywords:** multiplexed diagnostics, point-of-care testing, artificial intelligence (AI), signal deconvolution, nanomaterials

## Abstract

Decentralized diagnostics is undergoing a transformative shift from qualitative screening to high-precision quantification, driven by the clinical demand for rapid, point-of-care (POC) syndromic triage. Multiplexed lateral flow immunoassays (mLFIAs) serve as the foundational platform for this transition. However, their performance is limited by systemic factors such as fluidic lag, conjugate depletion, and spectral crosstalk. This review evaluates recent advances in engineered nanomaterials and artificial intelligence (AI)-driven detection as the dual pillars of next-generation multiplexing. The review covers different types of nanomaterial reporters—such as multicolor quantum dots, surface-enhanced Raman scattering nanotags, upconversion nanoparticles, surface-modified magnetic nanoparticles, and fluorescent nanodiamonds—that help address analytical challenges in lateral flow assays. We then discuss AI and machine learning methods, including convolutional neural networks, support vector machines, random forests, and transfer learning, that convert raw multi-channel signals into useful clinical data. Finally, we highlight the main challenges that still need to be addressed before these platforms can become WHO-ASSURED-compliant POC devices. The combination of engineered nanomaterial reporters and computational intelligence is transforming lateral flow assays into quantitative tools that can provide lab-quality clinical information at the POC.

## 1. Introduction

Over the past decade, decentralized diagnostics have undergone a significant transformation, moving from binary urine assays to fully quantitative analytical platforms. The conceptual origin of antibody-based diagnostics is traced to the radio-immunoassay (RIA) for human chorionic gonadotrophin (hCG) reported by Vaitukaitis and co-workers in 1972 [1], which established the antibody–antigen principle that later enabled the lateral flow immunoassay (LFIA) format. The lateral flow architecture itself, however, was described and patented in the late 1970s and early 1980s [2,3,4], and it was the commercialization of the home pregnancy LFIA that shifted the locus of diagnostics from the centralized laboratory to the home and established the modern concept of ‘point-of-care’ (POC) testing. A typical LFIA includes a sample pad, a conjugate pad loaded with antibody-functionalized reporter particles (usually colloidal gold nanoparticles, AuNPs), a nitrocellulose membrane with an analyte-specific test line and a control line, and an absorbent pad that moves the liquid by capillary action. The analyte–reporter complex travels from the conjugate pad to the test line, using either a sandwich format for large analytes or a competitive format for small molecules. The intensity of the test and control lines, which can be seen by eye or measured with an imager, shows the qualitative or semi-quantitative result. More detailed information about strip design, membrane chemistry, and conjugate preparation is available in previous studies [5,6,7].

Since its clinical debut, LFIA has proliferated globally as a robust, low-tech solution for resource-limited environments and a staple tool for practitioners in daily clinical workflows [8,9,10]. However, as healthcare increasingly emphasizes personalized medicine, particularly following the COVID-19 pandemic, the limitations of simple, single-target LFIAs have become increasingly apparent. Nowadays, clinical decision-making is no longer based solely on the detection of a single disease. Physicians must rapidly determine the underlying cause of a patient’s symptoms, a shift driven by the adoption of rapid multiplex syndromic panels that resolve overlapping clinical presentations [11,12]. For instance, when a patient presents with fever or respiratory distress, clinicians must distinguish between viral, bacterial, or co-infections. Reliance on single-marker testing is frequently insufficient, potentially resulting in delayed or inappropriate treatment. Consequently, multiplexed lateral flow immunoassays (mLFIAs), which enable simultaneous detection of multiple biomarkers, have become essential tools in clinical settings beyond traditional laboratory environments [13,14]. It is important to note that multiplex LFIAs have been used for years in routine testing. For example, multi-drug-of-abuse cassette tests on urine show that mLFIAs can be reliable when the analytes share a common matrix and similar analytical window. The new nanomaterial- and AI-enabled mLFIAs discussed here are not meant to replace these established qualitative tests. Instead, they expand the format to cover low-abundance, quantitative, and multi-matrix analytes that traditional colorimetric platforms cannot detect.

Despite their promise, expanding LFIA to several analytes raises a characteristic set of problems that we refer to here as the ‘multiplexing trade-off’: as the number of test lines and labels increases, the addition of further reagents introduces competing binding kinetics, optical or spatial cross-talk between neighboring lines, and systematic differences in capture efficiency between upstream and downstream zones owing to conjugate depletion and fluidic lag along the strip [13,14,15,16]. Similar phenomena have been described in detail by Bartosh et al. [15] and reviewed for infectious disease panels by Mohd Hanafiah et al. [16].

To address these challenges, recent advancements have focused on integrating novel nanomaterials with artificial intelligence (AI) [17]. Modern nanomaterials, such as quantum dots, upconversion nanoparticles (UCNPs), and surface-enhanced Raman scattering (SERS) tags, are engineered to produce distinct optical signals that are readily detectable by automated systems. Beyond these traditional optical reporters, quantum-enhanced nanomaterials—specifically fluorescent nanodiamonds (FNDs) containing nitrogen-vacancy (NV) centers—have emerged as a transformative solution for background-free sensing. These materials provide clear and unique spectral signatures, facilitating accurate computational analysis. Concurrently, AI has emerged as a critical component in enhancing assay performance. Advanced computational algorithms, such as convolutional neural networks (CNNs) or machine learning-driven signal deconvolution, can deconvolute overlapping signals, compensate for variations in fluid dynamics, and convert raw data into clinically actionable metrics. This paper reviews recent developments in the integration of nanotechnology and AI, highlighting their combined potential to enable rapid and high-precision quantitative analysis in clinical environments. While prior reviews have addressed nanomaterial labels for LFIA [18,19,20,21,22,23] or AI-assisted readout [17,24,25] individually, none have examined how these two engineering layers interact to collectively overcome the multiplexing trade-off—the fundamental trade-off between panel breadth and assay fidelity. This review is therefore organized around a reporter–reader co-design axis, framing nanomaterials as the signal encoding layer and AI as the signal decoding layer. Together, they constitute a dual-pillar architecture that redefines the upper performance limit of mLFIAs. We further benchmark current platforms against WHO-ASSURED criteria and regulatory SaMD frameworks, providing a forward-looking roadmap that prior catalogs have not offered.

## 2. Engineered Nanomaterials: The Building Blocks

The analytical capacity of a multiplexed assay is fundamentally limited by the physical and optical properties of its reporter probes. In recent years, the field has transitioned from passive colorimetric labels to “active” engineered nanomaterials designed specifically for high-fidelity digital interpretation. In LFIA, nanomaterials play two distinct roles that should not be conflated: (i) they act as carriers for antibody immobilization on the conjugate pad and (ii) act as detectable labels for immune complexes at the test line. The important properties of each nanomaterial depend on its role. For carriers, surface chemistry, colloidal stability, and antibody loading matter most. For labels, optical brightness, spectral specificity, magnetic moment, or spin contrast are key. In this review, the nanomaterial reporters are grouped into five types based on their physical signal-encoding mechanisms: (1) multicolor quantum dots (QDs), characterized by narrow Stokes-shifted emission; (2) surface-enhanced Raman scattering (SERS)-active composite nanotags, identified by vibration-specific Raman fingerprints; (3) upconversion nanoparticles (UCNPs), defined by anti-Stokes lanthanide light under near-infrared (NIR) excitation; (4) surface-modified magnetic nanoparticles (MNPs), distinguished by their magnetic moment and, when combined with a plasmonic shell, they enable dual-mode detection; and (5) quantum-enhanced fluorescent nanodiamonds (FNDs), recognized by spin-modulated nitrogen-vacancy (NV) center fluorescence. We specifically exclude classical AuNP-based colorimetric labels, which are covered elsewhere [9,10,18,19]; AuNPs are discussed only when they serve as plasmonic substrates in SERS or as a reference standard to compare against the newer reporters. Table 1 represents recent developments of nanomaterials as reporters for mLFIAs. These advanced materials act as the interface between the biochemical recognition event and the electronic readout, providing the signal density and stability required for quantitative syndromic triage [18].

### 2.1. Multicolor Quantum Dots

Although gold nanoparticles (AuNPs) are traditionally used for labeling conjugate probes, fluorescent reporters have demonstrated superior sensitivity and lower detection limits in LFIAs [19,53]. This gap is mainly due to the AuNP signal, which comes from a single, broad localized surface plasmon resonance. Its absorbance per particle is about one to two orders of magnitude lower than the total emission from a brightness-matched semiconductor nanocrystal [54]. On the other hand, Stokes-shifted fluorescence in fluorescent reporters makes it possible to use time- or wavelength-gated detection, which helps reduce scattered excitation light and membrane autofluorescence. Quantum Dots (QDs) are particularly promising due to their high molar extinction coefficients, quantum yields, and photostability [20,21]. Their broad absorption spectra and narrow, symmetric emission profiles facilitate precise antibody functionalization, supporting the development of highly sensitive diagnostic platforms. As a result, QD-based LFIAs typically achieve a 10–100-fold lower analytical LOD than the AuNP version of the same assay when the antibody pair, antigen, and matrix are matched [20,53]. QDs are particularly well-suited to multiplexing because their narrow, symmetric emission (FWHM < 30 nm) and broad single-wavelength excitation allow several non-overlapping colors to be excited from one source [20,21]. In addition to established applications in flow cytometry [55,56] and ELISA [57,58], multicolor QDs have expanded the capabilities of immunochromatography [59].

Faubert et al. developed a “Rainbow” LFIA capable of simultaneously detecting deoxynivalenol (DON), zearalenone (ZEN), and T2/HT2-toxins in barley [28]. The study optimized QD surfaces, demonstrating that silica-coated QDs with epoxy groups are superior for monoclonal antibody bioconjugation. Utilizing green, orange, and red QDs, the assay delivers results within 15 min and complies with European Commission legal limits. The system achieved a false-negative rate below 5%, indicating that multicolor QDs can yield reliable results without advanced laboratory infrastructure.

Recent progress has moved from using single quantum dots (QDs) to quantum dot nanobeads (QBs), which are tiny beads made of polymer or silica filled with hundreds of QDs for a stronger signal. Goryacheva et al. addressed the challenge of preserving fluorescence during the transition from organic solvents to aqueous media by engineering CdSe/CdS/ZnS core–shell heterostructures with tailored silica coatings, maintaining a quantum yield of 70% in water [29]. These silanized QDs enabled simultaneous detection of ZEN and DON in 34 naturally contaminated grain samples. The high brightness of these labels allowed the assay to meet stringent regulatory thresholds and demonstrated strong agreement with LC-MS/MS validation, underscoring the significance of shell engineering for probe stability. In clinical diagnostics, Wang et al. developed a three-dimensional (3D) tag consisting of multilayered QD nanofilms on graphene oxide (GO) nanosheets [26]. This configuration offers a larger reaction interface and higher QD loading compared to spherical beads. The resulting LFA biosensor facilitated simultaneous quantification of SARS-CoV-2, influenza A, and adenovirus. Signal amplification by the GO-QD film enabled ultrasensitive detection, such as 8 pg/mL for SARS-CoV-2 in saliva samples within 15 min, demonstrating potential for early-stage pandemic management.

**Figure 1:**
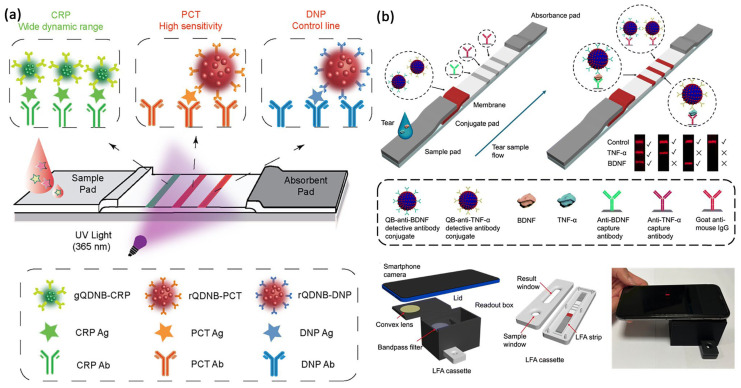
(**a**) Dual-color QDNB-based LFIA strips for the simultaneous detection of CRP and PCT. The detection antibody for CRP is labeled with smaller-sized g-QDNBs, while the detection antibody for PCT is labeled with larger-sized r-QDNBs. The detection antibody for DNP is labeled with r-QDNBs and functions as an independent control line. Reproduced with permission from Ref. [27]. Copyright 2024 American Chemical Society (License Number: 6241280830835). (**b**) Dual LFIA strip using QB-labeled antibodies as reporters for the detection of human BDNF and TNF-α in tears, read by a 3D-printed smartphone-coupled reader. The smartphone reader shown in (**b**) is the optical front-end that captures, normalizes, and transmits the QD fluorescence image to the downstream image-processing pipeline. Reproduced from Ref. [31] Copyright 2025 Royal Society of Chemistry under Creative Commons CC BY 3.0 license.

In a recent study, Duan et al. introduced a multiplex immunochromatographic assay (mICA) employing QBs [30]. Encapsulation of CdSe/ZnS QDs into polymer nanobeads produced distinct yellow, orange, and red signals for ZEN, ochratoxin A (OTA), and fumonisin B1 (FB1), respectively. This approach significantly enhanced signal intensity, achieving visual detection limits as low as 5 ng/mL for OTA. The strong correlation with ultra-performance liquid chromatography (UPLC) results supports the QB-mICA as a practical tool for rapid agro-food monitoring. Tang et al. solved a common testing problem: detecting one marker at high levels (C-reactive protein, CRP) while another is present in very small amounts (Procalcitonin, PCT) [27]. They used a mix of different colored and sized QD nanobeads—smaller ones for CRP to handle a wide range of amounts and avoid false results, and larger, brighter ones for PCT to spot even tiny amounts (Figure 1a). This method made a simple, one-step test that worked as well as lab tests and showed that QDs can be changed to fit medical needs. In eye care, Wu and team created a dual-test fluorescent test strip to measure glaucoma markers TNF-α and BDNF [31]. They coated QDs onto a silicon dioxide core to make nanobeads, which made the signal stronger. This allowed them to detect very low levels of the markers in tears (as low as 3.39 pg/mL for TNF-α and 4.13 pg/mL for BDNF). Using a 3D-printed readout box and a smartphone (Figure 1b), the test was more stable than standard lab tests and offered a portable, highly sensitive way to screen for early glaucoma.

Across the QD studies mentioned above, spectral multiplexing of 3–4 analytes is most reliably achieved when silica- or polymer-encapsulated cores are used, which help maintain quantum yield in water. Achieving sub-ng/mL LOD usually requires nanobead or 3D GO–QD structures for signal amplification, instead of single-particle labels. The main challenge is FRET-type spectral cross-talk from overlapping QD emission tails, but this is now better managed with ratiometric AI readout (see Section 3) rather than by making particles brighter. As a result, QD-LFIAs work best for multiplexed panels of three to four analytes in clear samples like saliva, tears, or serum, where autofluorescence from the matrix is moderate.

### 2.2. SERS-Active Nanotags

Surface-enhanced Raman scattering (SERS)-based LFIAs represent a significant advancement in POC diagnostics by replacing conventional colorimetric labels with specialized SERS nanotags [22]. These nanotags are composite structures comprising a plasmonic metal substrate, Raman reporter molecules (RRMs), and biological recognition elements. The vibrational fingerprint of the RRM creates very narrow Raman lines, with a typical FWHM of about 10 cm^−1^. This makes it possible to distinguish several spectral codes on a single test line. 5,5′-dithiobis (2-nitrobenzoic acid) (DTNB, 1330 cm^−1^) and 4-mercaptobenzoic acid (MBA, 1077 cm^−1^) are commonly used as RRMs because they form strong bonds with gold or silver surfaces through their thiol groups, have large Raman cross sections, and produce distinct, non-overlapping bands. The plasmonic substrate is most often AuNPs or AgNPs [60,61]. AuNPs offer chemical stability, low toxicity, and well-established surface chemistry, whereas AgNPs reach larger enhancement factors (up to ×10^9^) at the cost of oxidation and aggregation [62]. While AuNPs have already been used as conventional colorimetric reporters for LFIA, the SERS architectures described here introduce engineered composites around the AuNP. These include core–shell, gap-engineered, magnetically loaded, or rod-shaped nanoshells, designed to create dense electromagnetic hotspots and consistent Raman codes for multiplexing.

Recent research has focused on engineering novel nanostructures to maximize electromagnetic “hotspots” and improve stability through core–shell heterostructures and advanced surface engineering, thereby enabling the simultaneous detection of complex biomarker panels. Zhang et al. developed a Au@Ag core–shell SERS-LFIA functionalized with the standard DTNB and MBA reporters, dispensing six different hapten–protein conjugates across three test lines (two per line) for simultaneous detection of six major mycotoxins in maize within 20 min, with picogram-per-milliliter LODs for AFB1, ZEN, OTA, T-2, FB1, and DON, matching the accuracy of LC-MS [32]. Liu et al. fabricated SiO_2_@Ag nanotags carrying two layers of Raman dyes, achieving monodispersity and stability that allowed an ×800 sensitivity gain over the AuNP-LFIA baseline for anti-SARS-CoV-2 IgM/IgG, validated on 68 clinical sera [33]. Zhu et al. combined Au@SiO_2_ SERS tags with a nanostructured inverse-opal nitrocellulose membrane to reach fg/mL LODs for Aβ42 and Aβ40, 10-fold lower than standard SERS-LFA, demonstrating that the test-line membrane itself is an under-exploited engineering parameter [34].

**Figure 2:**
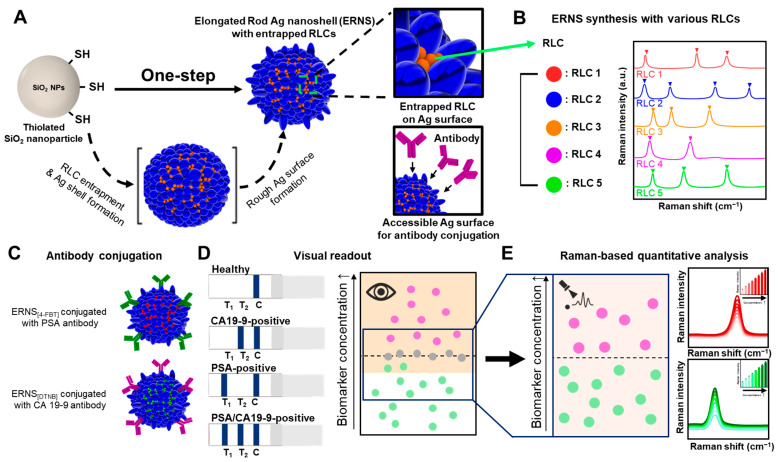
Dual-mode colorimetric-SERS lateral flow immunoassay (LFIA) utilizes elongated rod-shaped silver nanoshells (ERNSs) (**A**) as SERS nanotags. (**B**) ERNSs were synthesized with various Raman labeling compounds (RLCs) as nanoprobes for multiplex detection. (**C**) Antibody conjugated ERNS with different RLCs enable specific recognition of prostate-specific antigen (PSA) and carbohydrate antigen 19-9 (CA19-9). Application of ERNS-based probes to a SERS-LFIA platform, which allows for colorimetric (**D**) and Raman analysis (**E**), at the test lines provides quantitative detection, facilitating accurate sample classification and detection at ultralow concentrations. Reproduced from Ref. [38] under Creative Commons Attribution (CC BY) license.

In acute care, Jin et al. utilized gap-enhanced Raman tags (GERTs), which provide high photostability [35]. This platform enabled the simultaneous detection of respiratory viruses (SARS-CoV-2, FluA, and FluB) on a single test line with sensitivity enhanced by 2–3 orders of magnitude compared to traditional AuNPs. Quantitative analysis of cardiac troponin I (cTnI) reached 0.65 pg/mL, representing an order of magnitude greater sensitivity than fluorescent LFIA, which is essential for rapid onsite diagnosis of myocardial infarction. Shen et al. introduced a three-dimensional multi-hotspot membrane-like (ML) tag (MoDAu@Ag) by loading two layers of Au@Ag nanoparticles onto two-dimensional MoS2 nanosheets [36]. This design provided a large reactive interface and high stability. By incorporating three different Raman molecules into the gaps, “SERS encoding” was achieved, allowing for simultaneous quantitative detection of *P. aeruginosa*, *S. typhimurium*, and *E. coli* within a single test area. Tu et al. developed multilayered magnetic-core dual-shell nanoparticles (MDAu@Ag) featuring controllable nanogaps created via layer-by-layer assembly, resulting in numerous high-efficiency hotspots [37]. The magnetic Fe_3_O_4_ core enabled rapid capture and enrichment from complex samples. This dual-amplification strategy facilitated the simultaneous detection of four different veterinary drugs, including Kanamycin, ractopamine, clenbuterol hydrochloride, and chloramphenicol, with a 400-fold sensitivity increase over traditional methods. Another significant development involves elongated rod-shaped silver nanoshells (ERNS) with internally incorporated Raman labeling compounds (RLCs). Park et al. designed ERNSs as dual-mode tags, providing both a visual (colorimetric) signal for rapid screening and a strong SERS signal for precise quantification (Figure 2) [38]. When applied to cancer biomarkers (PSA and CA19-9), the ERNS-based LFIA achieved LODs approximately 500–685-fold below the established clinical decision thresholds, enabling early-stage cancer detection at sub-clinical concentrations where standard colorimetric LFIAs would fail to produce a visible signal. This dual-mode approach bridges the gap between simple qualitative field tests and high-sensitivity laboratory diagnostics.

Together, these advancements show that SERS-LFIAs have the highest spectral channel count among current LFIA reporter families (three to six codes per test line) and the lowest reported limits of detection (fg to pg/mL). However, they always need a portable or benchtop Raman spectrometer for readout. The main analytical challenge is spectral mixing between nearby RRM peaks and background fluorescence from the sample, but AI-based deconvolution can now address both issues. We believe SERS-LFIAs work best for high-stakes panels that need laboratory-level sensitivity and can use an instrumented reader, such as tests for cancer biomarkers, mycotoxins, veterinary drug residues, or low-abundance respiratory antigens.

### 2.3. Upconversion Nanoparticles

Upconversion nanoparticles (UCNPs) represent a distinct class of luminescent nanomaterials that have significantly advanced the capabilities of multiplexed LFIA [23]. Unlike fluorescent reporters—such as organic dyes, carbon dots, or QDs—which follow a standard Stokes shift by emitting longer-wavelength light upon high-energy excitation, UCNPs operate through an anti-Stokes process. These lanthanide-doped crystals sequentially absorb two or more low-energy near-infrared (NIR) photons and convert them into high-energy visible or ultraviolet (UV) light. The upconversion mechanism, primarily governed by energy transfer upconversion (ETU) and excited-state absorption (ESA), provides a significant advantage for diagnostics by eliminating autofluorescence. Since biological matrices do not emit light under NIR excitation, UCNPs enable an exceptionally high signal-to-noise ratio and facilitate deeper tissue penetration. The multiplexing capability of UCNP-based LFIA is primarily achieved through spectral encoding or the use of multiple spatial test zones. Precise modulation of the type and concentration of lanthanide dopants, such as Er^3+^, Tm^3+^, Ho^3+^, and Yb^3+^, within the crystal matrix enables fine tuning of luminescence emission across the electromagnetic spectrum [23,63]. Specific dopant combinations produce distinct, non-overlapping emission peaks ranging from blue and green to orange and red, all under a single 980 nm NIR excitation source. For example, studies have shown that adjusting the ratios and combinations of Er^3+^, Tm^3+^, and Yb^3+^ allows for UCNP emission colors to be tuned from yellow to red or from blue to white [64]. This property of single-wavelength excitation and multi-wavelength emission permits different-colored UCNPs to be conjugated to specific antibodies, enabling simultaneous detection of multiple targets on a single LFIA strip without spectral interference or the requirement for complex multi-laser optical systems.

Recent studies have demonstrated that UCNP-based LFIAs for targets such as SARS-CoV-2 can achieve limits of detection as low as 3.56 pg/mL, a 100-fold improvement over traditional AuNPs, with a linear range of 0.01–100 ng/mL [65]. Applications of UCNPs exhibit superior performance in LFIA systems for the detection of various biomarkers, such as mycotoxins [66], pathogens [40,67], prostate-specific antigen (PSA) [68], C-reactive protein (CRP) [69], and circulating plasma FKBPL/CD44 protein from individuals with early-onset preeclampsia (EOPE) [70]. Jin et al. developed a lateral flow aptamer assay that simultaneously detects three targets: mercury ions (heavy metals), ochratoxin A (small-molecule mycotoxins), and *Salmonella* (bacteria) [40]. By functionalizing multi-colored UCNPs with specific aptamers, separate color channels were utilized to prevent cross-reactivity. The assay achieved impressive detection limits (5 ppb for Hg^2+^, 3 ng/mL for OTA, and 85 CFU/mL for Salmonella) within 30 min. A smartphone-based readout was used instead of a bulky CCD camera, highlighting the transition of UCNP-LFIA from the laboratory to a portable, POC platform.

**Figure 3:**
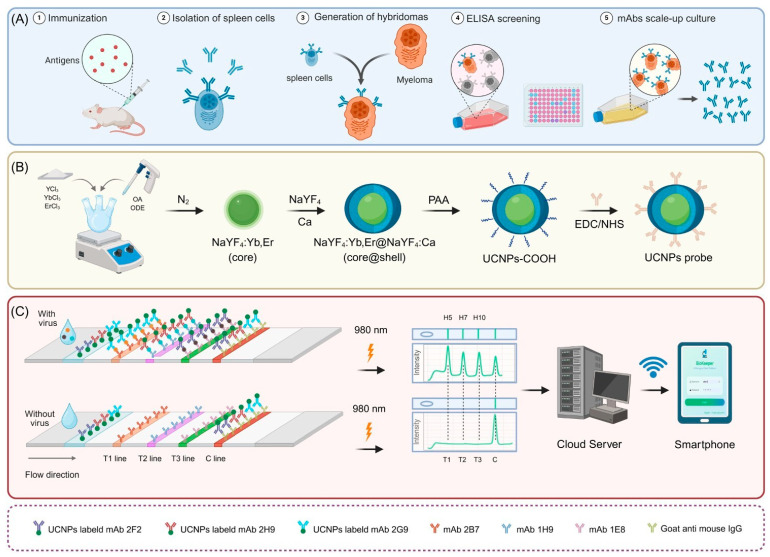
Smartphone-assisted core–shell UCNP-LFA for simultaneous detection of H5, H7, and H10 avian influenza viruses. (**A**) Monoclonal antibodies are produced via hybridoma technology. (**B**) Fluorescent probes prepared using core–shell UCNPs. (**C**) UCNP-LFA strip read on a smartphone, with T1, T2, and T3 indicating test lines for H5, H7, and H10, respectively; C line functions as an internal control. Reproduced from Ref. [39] under Creative Commons Attribution (CC BY 4.0) license.

To address the complexity of foodborne pathogens, Zhao et al. developed a 10-channel up-converting phosphor technology lateral-flow disk that integrates ten optimized single-target strips on one rotating cartridge for the simultaneous screening of *E. coli*, *Listeria*, *Vibrio*, and seven other pathogens, with 100% concordance to culture across 279 food samples in 20 min. He et al. designed a disc-format LFIA with green core–shell UCNPs that quantifies the inflammatory triad MMP-8, IL-1β, and TNF-α in gingival crevicular fluid for chair-side periodontitis monitoring, with up to 0.995 correlation against clinical laboratory assays [42]. Zhang et al. reported a significant advancement in zoonotic disease surveillance by developing an intelligent, smartphone-integrated LFA for the highly pathogenic H5, H7, and H10 subtypes of Avian Influenza Viruses (AIVs) [39]. By employing core–shell UCNPs conjugated to subtype-specific monoclonal antibodies targeting haemagglutinin (HA) proteins (Figure 3), the platform achieved ultra-sensitive detection limits of 0.0156 ng/mL for the H7 subtype, with zero cross-reactivity against other respiratory pathogens. Rigorous validation using 260 human and avian clinical samples demonstrated 100% concordance with gold-standard real-time RT-PCR, while the integration of a smartphone analytical interface enabled automated quantification and cloud-based data sharing within a 10-min assay window. Ultimately, UCNPs provide the highest signal-to-background ratio among current LFIA reporters because their excitation occurs in a biologically silent NIR window. The main challenge is their lower brightness per particle, but this can be improved with core–shell shielding, optimized lanthanide ratios, and AI-based ratiometric readouts that use color separation as a quantitative signal. We find that UCNP-LFIAs work best for detecting targets in optically dense or autofluorescent samples like blood, food, or gingival crevicular fluid, as well as for pathogen screening and multi-class panels that include inorganic, organic, and microbial targets.

### 2.4. Surface-Modified Magnetic Nanoparticles

Magnetic nanoparticles (MNPs) offer a different method from labels that use optical properties. Instead of colorimetric or fluorescent reporters, MNP-based LFIA measures magnetic signal intensity, which is not affected by the sample matrix. Labels like Fe_3_O_4_ work well in biological solutions with very low residual flux density, enabling higher sensitive than optical sensing methods. For example, Wang et al. developed a highly sensitive atomic magnetometer-based biosensing platform that uses linear scans to measure residual flux density and locate MNP-tagged test strips [71]. Their system achieved an LOD of 0.01 ng/mL for carcinoembryonic antigen (CEA), making it about 100 times more sensitive than traditional LFIA. In addition to higher sensitivity for detecting small amounts of biomarkers, magnetic readout provides a digital signal that can be directly linked to analyte concentration.

Beyond signal transduction, MNPs are also exploited as immunomagnetic separation agents [72]. Wang et al. developed magnetic quantum dot (MagQD) nanoparticles by attaching fluorescent QDs to Fe_3_O_4_ cores [43]. This allowed them to enrich and detect the toxic proteins BoNT/A and SEB in milk and juice, reaching detection limits as low as 2.52 pg/mL and 2.86 pg/mL, respectively. To address the problem of dark-colored foods like grape juice, Hao et al. designed magneto-gold nanohybrids (MGNHs) that use magnetic separation to remove interfering pigments before giving a clear colorimetric result [73]. This method achieved a detection limit of 0.094 ng/mL, similar to LC-MS accuracy. Chen et al. also developed fluorescent ZnCdSe/ZnS magnetic quantum dot nanocomposites (MQNs) to detect streptomycin and dihydrostreptomycin in milk and animal tissues [44]. By combining the MQN’s strong signal amplification with magnetic pre-concentration, their platform was up to 42 times more sensitive than earlier tests. Together, these advances show that combining magnetic separation with advanced optical signals offers a reliable and sensitive way to quickly measure trace chemical and biological contaminants.

In another approach, recent magnetic LFIAs have introduced new diagnostic tags, such as Au-Fe_3_O_4_ nanostars/nanoclusters and Janus particles [74,75]. These nanostructures combine noble-metal plasmonic features with superparamagnetic MNPs. By using both optical labels and magnetic nanoparticles, researchers have developed a dual-readout system that uses magnetism and plasmonics. This design makes it possible to enrich and purify target labels from larger sample volumes, such as 5 mL of whole blood or food, before applying them to the membrane. This pre-concentration step significantly lowers the detection limit by increasing the amount of target analyte in the sample pad. The dual method also allows for quantitative results using magnetic detection. Even if the analyte is too low to see by eye, digital sensors can still measure it using magnetic or photothermal signals [76]. Wen et al. developed a multi-modal capability mLFIA for simultaneous screening of respiratory viruses, including H3N2 influenza and SARS-CoV-2 [45]. By synthesizing near-infrared (NIR)-responsive Janus Au_shell_-Fe_3_O_4_ nanoparticles, a platform supporting both colorimetric and photothermal detection enables two distinct test lines to capture virus–nanoparticle complexes, producing visible bands for rapid qualitative screening and high-sensitivity photothermal signals under NIR laser irradiation. Photothermal detection covered a range from 10 to 106 pg/mL, with limits of detection of 2 pg/mL for H3N2 and 7 pg/mL for SARS-CoV-2. This method is about 10,000 times more sensitive than visual detection and offers a fast way to detect viruses early in clinical samples. Among the mentioned reporter families, only MNP-based LFIAs offer a readout that is naturally unaffected by the sample matrix, and they allow for easy pre-concentration from large sample volumes. However, the main challenge is the need for specialized instruments for quantification. This requirement restricts their use to dedicated POC settings. As a result, these platforms are best suited for detecting opaque or particulate samples, like whole blood, milk, fruit juices, or environmental water, and for situations where pre-enrichment is indispensable.

### 2.5. Quantum-Enhanced Probes: Fluorescent Nanodiamonds

Fluorescent nanodiamonds (FNDs) are an emerging class of quantum-enhanced probes for LFIA, offering superior photostability and sensitivity compared to traditional organic dyes or quantum dots [77,78]. These carbon-based nanomaterials derive their special optical properties from nitrogen-vacancy (NV) centers, point defects in the diamond lattice that emit strong, stable fluorescence. This fluorescence does not fade or blink, even under strong, continuous light. FNDs are also highly biocompatible, chemically stable with various oxygen-containing groups such as −COC–, −COH, and −COOH after extensive acid washes, making it easy to attach antibodies to their surfaces while maintaining a high quantum yield in complex biological samples. One of the biggest advantages of FNDs is the ability to selectively modulate the signal for background-free detection. When an external microwave or magnetic field is applied, the fluorescence from NV centers can be controlled, enabling separation of the target signal from the natural autofluorescence from the nitrocellulose membrane or complex biological samples [79,80]. As a result, FND-based LFIAs can detect concentrations much lower than those of standard colorimetric assays, making them a strong platform for early disease diagnosis and for detecting very small amounts of biomarkers.

**Figure 4:**
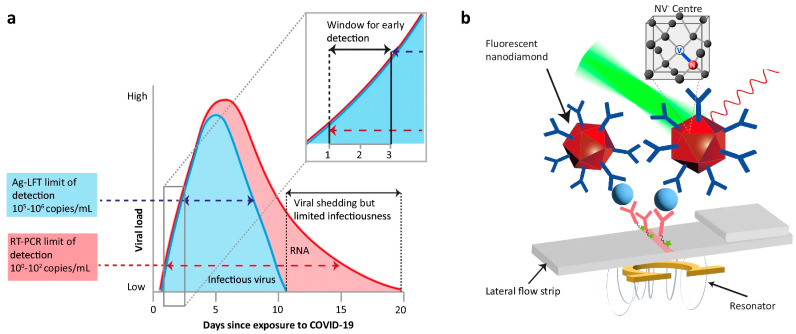
Quantum-enhanced FND-enabled LFIA for SARS-CoV-2 detection via microwave-assisted detection. (**a**) The infection timeline compares RNA and infectious virus levels to the RT-qPCR detection limit (10^2^ copies/mL). The highlighted area shows a 1 to 3-day period when better antigen-detecting lateral flow tests (Ag-LFT) sensitivity allows earlier detection. (**b**) The diagram shows the FND–antibody sandwich complex at the test line. An omega-shaped resonator at 2.87 GHz changes the spin state population, creating a time-varying fluorescence signal. Reproduced from Ref. [88] under Creative Commons Attribution (CC BY 4.0) license.

For instance, Hui et al. utilized a periodic, time-varying field of 30 mT to achieve a modulation depth exceeding 10% [79]. By integrating this property with a lock-in detection method, the researchers successfully isolated the FND signal from background signals and sample autofluorescence, reaching a detection limit against FND of 0.04 ng/mm^2^ on nitrocellulose membranes and 1 ng/mL (approximately 1 fM) in aqueous solutions within a 10-s acquisition window. Applying this technique, FND-based LFIAs are able to detect various biomarkers, such as human chorionic gonadotropin (hCG), C-reactive protein (CRP) [81], p-tau protein [82], interleukin-6 [83], ESAT6 (6 kDa early secretory antigenic target) of Mycobacterium tuberculosis [84], influenza A virus reference gene 6 (RG6) [85], Dengue virus NS1 proteins [86], and SARS-CoV-2 N and S proteins from different variants [87]. Taking another approach, Miller et al. pioneered the application of FND-based LFIA through microwave-assisted detection to modulate the emission intensity of FND labels and used frequency-domain analysis to separate the signal (Figure 4) [80]. This allowed them to reach a detection limit of 8.2 × 10^−19^ M for a biotin–avidin model, which is 100,000 times more sensitive than traditional AuNPs. With this high sensitivity, they could identify single-copy HIV-1 RNA, even in complex clinical plasma samples. Building on this approach, DeCruz et al. tested a spin-enhanced nanodiamond method for detecting SARS-CoV-2 antigens in 103 respiratory swab samples [88]. Compared to RT-qPCR, the FND-based test showed 95.1% sensitivity and 100% specificity, with no cross-reaction to other common respiratory viruses. Patient data modeling showed that this quantum-enhanced method could find the virus about two days earlier than standard rapid tests and detect more than twice as many patients on the first day of symptoms [88]. Together, these studies show that FNDs deliver the lowest analytical LODs of any current LFIA reporter (down to the attomolar scale) but require a microwave or magnetic-modulation reader. However, further developments are essential to realize its application in the multiplex format of LFIAs.

Taken together, the studies reviewed in this section indicate that nanomaterial reporters have not merely competed for identical applications but have instead established distinct deployment niches, each defined by a dominant physical or optical property. Table 2 summarizes this perspective by listing, for each reporter family, the application classes in which it is most frequently utilized in the cited studies, the property most commonly cited as the rationale for selection, and the principal bottleneck currently limiting broader adoption. Multicolor QDs are predominantly selected for spectrally coded food-safety and acute-care panels where three to five analytes must be resolved on a single test line. SERS nanotags are preferred for low-abundance clinical panels requiring sub-AuNP LODs and five or more spectrally orthogonal barcodes. UCNPs are favored for autofluorescent food and environmental matrices, where anti-Stokes excitation eliminates matrix background. Surface-modified MNPs are chosen for pathogen and biomarker detection in complex biofluids, where magnetic pre-enrichment decouples sensitivity from capillary-flow residence time. FNDs occupy a narrower niche, serving ultrasensitive assays in which NV centers photostability and optically detected magnetic resonance (ODMR)-based background rejection are decisive. Overall, the performance of an mLFIA is determined by the alignment between reporter chemistry, analytical challenge, and readout hardware, rather than by any single attribute of the reporter. This observation motivates the next section, which explores how AI-driven detection strategies are being co-developed with these reporters to deconvolute multi-channel signals into reliable quantitative outputs.

## 3. AI-Driven Detection in mLFIAs

### 3.1. Reporter–Reader Combinations

The integration of artificial intelligence (AI) and machine learning (ML) into LFIA represents a significant shift from subjective, qualitative assessments to more reliable digital diagnostics [24]. Although traditional LFIAs offer portability, they are susceptible to human observer bias, particularly when faint or ambiguous bands complicate result interpretation [89]. This problem scales unfavorably with multiplexing: the human eye cannot reliably resolve three to five spectrally overlapping test lines, nor can it correct for flow-induced temporal drift or for hardware differences between smartphone cameras. ML-based pattern recognition addresses these limitations with algorithms whose inductive biases match specific LFIA failure modes. Convolutional neural networks (CNNs) learn color- and shape-level features that resolve faint or chromatically overlapping test lines [25,90]; support vector machines (SVMs) classify feature vectors built from peak intensity and bandwidth using linear or kernel decision boundaries; random forests aggregate decision trees to suppress pixel-level noise and overfitting; long short-term memory (LSTM) networks exploit the temporal evolution of the strip image to normalize for fluidic lag and enable kinetic read-out [90]; and transfer learning and vision transformers (ViTs) generalize across cameras and lighting conditions, compensating for hardware disparity in smartphone-based readout.

Set against the reporter landscape described above, these algorithms are not interchangeable; each is specifically matched to a reporter family based on its primary performance bottleneck. For multicolor QDs, spectral cross-talk is the main constraint, so ratiometric or CNN-based deconvolution of overlapping emission bands might be used [25]. The same CNN feature extractor could reduce the residual matrix autofluorescence, while transfer learning or ViT-based calibration is applied when assays are imaged across different smartphones [91,92]. For SERS nanotags, spectral cross-talk occurs in the Raman domain and is addressed by CNN, partial least squares (PLS), or ratiometric spectral deconvolution of the narrow vibrational fingerprints [93]. Since the Raman band is outside the autofluorescence envelope, the main concern is hot-spot variability, which is filtered using random forest or SVM classifiers [94]. For UCNPs, anti-Stokes emission rejects matrix autofluorescence, shifting the bottleneck to dynamic range and hardware variability. The low quantum yield makes smartphone capture challenging, so transfer learning or ViT pipelines are commonly used [95], with CNN-based color unmixing for parallel lanthanide emissions and supported by CNN-based color unmixing when two or more lanthanide emissions are read in parallel [96]. For MNP strips, the magnetic channel avoids optical cross-talk, but performance is limited by non-equilibrium enrichment and capillary flow. LSTM-based temporal normalization of the accumulation curve is typically used, with random forest regression on the post-enrichment signal enabling quantification [97,98]. As a result, when spectral or fluidic cross-talk is the limiting factor, improving the absolute LOD of a multiplex LFIA requires advances in the reader, not simply a brighter reporter.

### 3.2. Applications of AI/ML Readout Strategies in LFIA

**Figure 5:**
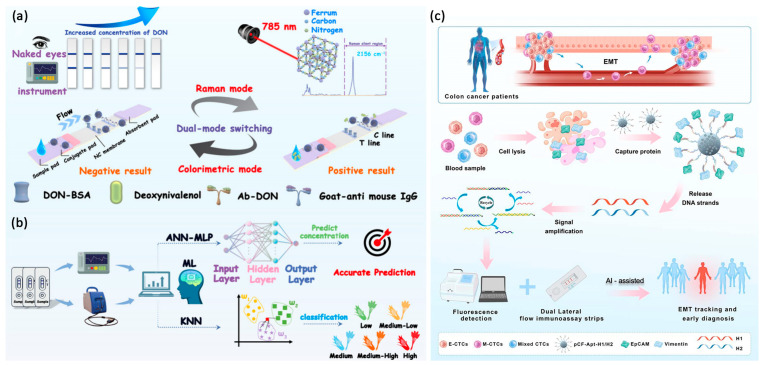
(**a**,**b**) Colorimetric-SERS dual-mode LFI (**a**) and machine learning integration for optimized colorimetric and SERS analysis of deoxynivalenol (DON) in grain (**b**). Reproduced with permission from Ref. [93] Copyright 2025 American Chemical Society (License Number: 6241260833952). (**c**) Multiplex lateral flow immunoassay integrates machine learning for sensitive colon cancer diagnosis. Reproduced from Ref. [97] under Creative Commons CC BY-NC-ND 4.0 license.

Recently, many studies have shown that combining LFAs with ML algorithms can improve sensitivity, specificity, and objectivity. Table 3 summarizes recent developments of AI and ML integrated LFIAs. This approach has led to the creation of highly accurate diagnostic systems for both laboratory and field use. Huang et al. created a portable fluorescence reader using UCNP-labeled LFIA to quickly measure methamphetamine levels [96]. At first, the system used the standard T/C intensity ratio, but environmental noise made it hard to detect very low concentrations (below 0.1 ng/mL). To solve this, they used a CNN model to identify image features across low methamphetamine levels (0 to 0.5 ng/mL). This deep learning method could distinguish between weakly positive and negative samples with 92% accuracy, demonstrating that CNNs can capture important diagnostic details that traditional ratio analysis might miss. Wang et al. extended this approach to IoT-class devices using transfer learning: large pretrained CNN backbones (ResNet50, VGG16, GoogleNet, MobileNet V2, etc.) were fine-tuned with a small UCNP image set, reaching almost 100% quantitative accuracy on noisy images and removing the need for complex preprocessing [95]. Sun et al. combined a colorimetric/SERS dual-mode strip with k-NN and ANN classifiers (Figure 5a,b), exploiting a Prussian-blue Raman peak at 2156 cm^−1^ in the biologically silent region; the ML pipeline reached an LOD of 4.21 pg/mL for DON, 37-fold below the AuNP baseline, with 98.8% classification accuracy and R^2^ = 0.993 quantitative correlation [93]. Across these studies, the AI layer is responsible for an approximately 5–40-fold improvement in usable LOD over the same hardware read with a conventional T/C ratio; importantly, several of the same tasks (background subtraction, perspective correction, and white-balance normalization) can also be solved with classical image-processing pipelines, and the AI-attributable gain is therefore best interpreted as the marginal gain on top of strong-baseline image preprocessing rather than as the gain over raw input.

In mLFIAs, AI plays a bigger role because overlapping signals and multiple test lines can lead to more analytical errors. He et al. developed a liquid biopsy platform that combines entropy-driven signal amplification with ML to simultaneously detect the EMT biomarkers EpCAM and Vimentin [97]. Their method used an anti-fouling magnetic probe to capture circulating tumor cells (CTCs), then a series of strand-displacement reactions produced many DNA signaling molecules for colorimetric detection in a AuNP-based mLFIA (Figure 5c). This multi-step amplification enabled very low detection limits of 0.22 ng/mL and 0.16 ng/mL for EpCAM and Vimentin, respectively, allowing for tracking of EMT processes at concentrations as low as 10 cells/mL. Using ML algorithms such as SVM, random forest, logistic regression, and XGBoost to classify dual-target intensities, the platform can accurately distinguish healthy individuals from colon cancer patients, demonstrating its promise as a clinical diagnostic tool. In another study, Vdokaki et al. used deep learning to address the challenges of single-nucleotide polymorphism (SNP) genotyping in *Olea europaea*, where allelic variants differ by just one base pair [104]. Their process included a multiplex PCR step and a 15-min multiallelic lateral flow reaction, analyzed by CNN and decision tree models; the CNN reached 97% accuracy on real olive oil and leaf samples. Together, these studies confirm that ML transforms mLFIAs into smart analytical tools whose sensitivity and dynamic range cannot be matched by hardware alone.

## 4. Challenges and Future Perspectives

Moving mLFIA from the lab to clinically validated quantitative tools means overcoming several analytical challenges. While the theory behind high-dimensional detection is well known, mLFIA’s real-world performance relies on how different binding events and biological samples interact. To get accurate, lab-quality results from what starts as a qualitative test, strong methods are needed to minimize errors that come from measuring multiple analytes. In this review, we group these challenges into four main categories: fluidic and matrix, signal-encoding, computational, and clinical-translation limitations.

### 4.1. Fluidic and Matrix Limitations

A significant obstacle is the multi-variant matrix effect, in which interference from serum proteins, lipids, or mucins occurs through both physical rheology and chemical cross-talk [16]. Mathematically, capillary flow on nitrocellulose can be described by the Lucas–Washburn relation, which shows that capillary flow velocity is inversely proportional to fluid viscosity and directly proportional to membrane pore radius [105,106]. This is important because matrix viscosity and pore swelling under sample loading can vary by 10–40% in real clinical samples. These changes cause uneven capture between upstream and downstream test lines and lead to the typical ‘fluidic lag’ seen in multiplex strips. As a result, downstream test lines become more prone to timing errors. Fluidic lag is a predictable source of bias and has been addressed by using hardware solutions like laminar wicking pads or calibrated control lines [5,107], or by applying ML-based temporal normalization that uses wicking kinetics as a reference [25,102].

### 4.2. Signal-Encoding and Quantification Limitations

In high-density signal environments, where multiple colorimetric, fluorescent, or SERS-active reporters are concentrated on a single 5 mm strip, traditional linear regression models often fail to provide accurate results. In this aspect, AI-based spectral deconvolution might help to resolve overlapping emission or Raman peaks: CNNs can learn the spatial and color patterns of overlapping fluorescence tails directly from RGB or multispectral strip images [99,108], while PLS, non-negative matrix factorization (NMF), or independent component analysis (ICA) can separate hyperspectral SERS data into distinct nanotag fingerprints [93,94]. Ratiometric (T/C) analysis can in turn be replaced by multivariate regression methods, such as random forest, ridge regression, or shallow neural networks, which can be applied to the full set of test- and control-line intensities [97,98]. This approach captures cross-channel coupling as a signal, rather than treating it as noise, as the univariate T/C ratio does.

Moreover, the four-parameter logistic (4PL) model, often cited as the ‘global standard’ for quantitative LFIA, is a symmetric-sigmoid calibration that fits both competitive and sandwich immunoassays when the dose–response is symmetric; asymmetric curves—common at the high-dose end of sandwich assays—are better served by a five-parameter logistic (5PL) fit or empirical calibration [109]. Manual interpretation of multiple test zones is both time-consuming and subjective, but a substantial part of the bias can already be removed by inexpensive non-AI solutions: cassette reference markings, dot-line registration, white-balance correction, and dark-field shielding are widely deployed in commercial multiplex tests [5,107]. AI is most needed where these classical fixes have already been exhausted, specifically for (i) spectral deconvolution of overlapping reporter channels [108], (ii) fluidic temporal normalization of asynchronous wicking [102], and (iii) cross-device generalization across smartphone cameras and lighting conditions, which is increasingly addressed by transfer learning and vision transformers (ViTs) rather than by per-device recalibration [91,92].

### 4.3. Computational Limitations

Although AI has the potential to overcome many of the limitations of mLFIAs, moving AI-integrated LFIAs from lab prototypes to clinically validated tools requires bridging the gap between controlled experiments and the unpredictable conditions found in real-world use. Table 4 lists the main challenges in implementing AI-LFIA across the data, hardware, regulatory, and clinical-translation domains addressed in this and the next subsection. A key issue is data integrity, since most models are trained on small, ideal datasets that do not reflect real sample differences, like changes in blood viscosity or hemolysis, or mistakes made by users, such as using the wrong sample volume or affecting fluid flow. Technical and environmental factors, like differences in smartphone cameras and lighting, can also change how test bands appear, leading to unreliable results. Three strategies have emerged to close this generalization gap. First, training-set augmentation with intentionally noisy or matrix-spiked images and synthetic-data generation by GANs or diffusion models exposes the model to the long tail of real conditions before deployment [17]. Second, on-strip color-calibration patches give the algorithm a per-image reference for white balance and exposure, standardizing readouts across device generations without per-device retraining. Third, transfer learning and vision transformers (ViTs) are increasingly used to inherit feature representations learned on large public image datasets and fine-tune them on small clinical sets [91,92].

### 4.4. Regulatory and Clinical-Translation Limitations

Bringing AI-integrated LFIAs to the clinic requires meeting the regulatory standards already applied to laboratory-grade in vitro diagnostics, and then earning the clinician and patient trust on which routine adoption ultimately depends. On the regulatory side, AI-driven readout falls under the Software-as-a-Medical-Device (SaMD) framework recognized by the FDA, EU MDR/IVDR, and IMDRF, which classifies the algorithm itself as a regulated device and requires a documented quality-management system (e.g., ISO 13485) and risk-management process (e.g., ISO 14971). A challenge specific to AI-LFIA is the handling of ‘adaptive’ or ‘continuously learning’ algorithms, where retraining or model drift can occur after market release: regulators have responded with Predetermined Change Control Plans (FDA, 2024) and analogous instruments under the EU AI Act, which require manufacturers to declare in advance which model parameters are allowed to evolve, on what data, and under what monitoring regime. Locked-algorithm approval remains the lowest-friction route to market, but it forecloses the very generalization strategies—continual transfer learning and federated updates—that AI-LFIA depends on for real-world robustness, and the resolution of this tension is one of the dominant outstanding regulatory questions for the field.

Even a regulator-approved AI-LFIA must still earn clinician and patient trust before it is adopted in routine workflow. The main obstacle is that deep-learning models are often hard to interpret, making clinicians hesitant to rely on results they cannot audit. Explainable-AI (XAI) tools are therefore essential, with two methods carrying most of the practical weight today: saliency/Grad-CAM heatmaps that show which region of the strip drove the call, and per-result confidence scores that let the clinician triage low-confidence cases for laboratory confirmation rather than accept a brittle binary output [17]. Beyond making results explainable, decentralized use of AI-LFIAs needs infrastructure that traditional lab systems do not. This includes cloud connectivity for updating models across many users, federated learning so hospitals can help retrain models without sharing patient data, and on-device transfer learning for places with limited internet access.

The next generation of multiplex LFIAs is emerging where nanomaterial reporter development meets computational intelligence, and it is the disciplined pairing of these two areas, rather than either one alone, that can bring together the speed of rapid POC tests and the accuracy of clinical laboratory instruments. This combination also helps the platform meet the WHO-ASSURED criteria (Affordable, Sensitive, Specific, User-friendly, Rapid and Robust, Equipment-free or minimal, and Deliverable to end-users) and provides a strong path toward reliable, decentralized diagnostic tools for real-time disease monitoring and precision medicine.

## 5. Conclusions

mLFIAs have evolved from simple screening strips to advanced analytical platforms, marking a major change in decentralized diagnostics. By moving beyond the limitations of linear paper strips, new multiplexing designs use 2D layouts and spectral methods, enabling the complexity required for modern syndromic triage. The combination of engineered nanomaterials, such as QDs, UCNPs, SERS tags, and FNDs, with AI-based pattern recognition has helped overcome past challenges, such as spectral overlap and slow fluid flow. Now, we are using ML to convert complex biochemical signals into clinically useful information, going beyond what the human eye can see. As these technologies develop, next-generation multiplex assays will become more than just detection tools—they will be key to global health security, offering lab-quality precision in a portable format.

## Figures and Tables

**Table 1:** Nanomaterial-enabled multiplex lateral flow immunoassays.

Color Probe/Reporter	Multiplexing Mode	Target Analytes	Sample Matrix	Analytical Sensitivity (LOD)	Assay Time	Reader	Ref.
QDs—CdSe@ZnS-COOH on GO film	Spatial (3-line)	SARS-CoV-2 N; Influenza A virus; Human adenovirus	Buffer, saliva	8 pg/mL; 488 copies/mL; 471 copies/mL	15 min	Fluorescent reader	[26]
QDs—dual-color QDNBs	Spatial + Spectral	CRP; PCT	Buffer, plasma	0.1 ng/mL; 0.09 ng/mL	15 min	Fluorescent reader	[27]
QDs—silanized core/shell	Spatial + Spectral	DON; ZEN; T-2/HT-2	Barley	below EC legal limits †	15 min	Colorimetric, Fluorescent reader	[28]
QDs—silica-encapsulated	Spatial + Spectral	ZEN; DON	Maize	regulatory threshold met †	15 min	Fluorescent reader	[29]
QDs—CdSe/ZnS QDNBs	Spatial + Spectral	ZEN; OTA; FB1	Maize	10/5/20 ng/mL	10 min	Fluorescent reader	[30]
QDs—SiO_2_@D-QD	Spatial	TNF-α; BDNF	Tears	0.39/4.13 pg/mL	n.r.	3D-printed/smartphone	[31]
SERS—Au@Ag core–shell + DTNB/MBA	Spatial + spectral (3 lines/2 dyes)	AFB1; ZEN; FB1; DON; OTA; T-2	Maize	0.96/6.6/260/110/15.7/8.6 pg/mL	20 min	Portable Raman	[32]
SERS—SiO_2_@Ag dual Raman dye	Spatial	SARS-CoV-2 IgM; IgG	Serum	n.r. (×800 vs. AuNP)	25 min	Portable Raman	[33]
SERS—Au@SiO_2_	Spatial	Aβ42; Aβ40	Serum	15.3; 16.8 fg/mL	20 min	Benchtop Raman	[34]
SERS—Gap-Enhanced Raman tags (GERTs)	Spectral (single-line, 3 codes)	SARS-CoV-2 S protein; FluA; FluB	n.r.	1.26 pg/mL (SARS-CoV-2)	15 min	Portable Raman	[35]
SERS—MoDAu@Ag (3D ML tag)	Single-zone spectral encoding	*P. aeruginosa*; *S. typhimurium*; *E. coli* O157:H7	Bacterial spike	30–40 cells/mL	14 min	Portable Raman	[36]
SERS—MDAu@Ag layered nanogap	Single-line, 4 codes	Kanamycin; ractopamine; clenbuterol; chloramphenicol	Animal-derived food	0.52/2.5/0.87/6.2 pg/mL	35 min	Portable Raman	[37]
SERS—Elongated rod-shaped Ag nanoshells (ERNS)	Dual-mode spatial (colorimetric + SERS)	PSA; CA19-9	Serum	8.0 pg/mL; 54 mU/mL	20 min	Colorimetric + Raman	[38]
UCNPs—core–shell (3-color)	Spectral (3-color)	H5/H7/H10 AIV	Avian/clinical (*n* = 260)	0.0156–0.0625 ng/mL	10 min	Smartphone	[39]
UCNPs—multicolor + aptamers	Spectral (3-color)	Hg^2+^; OTA; Salmonella	Spiked water/food	5 ppb/3 ng/mL/85 CFU/mL	30 min	Smartphone	[40]
UCNPs—NaYF_4_:Yb,Er disk (10-channel)	Spatial (10-channel)	*E. coli* O157:H7, S. paratyphi A, S. paratyphi B, S. paratyphi C, S. typhi, S. enteritidis, S. choleraesuis, V. cholera O1, V. cholera O139, and V. parahaemolyticus	Food (*n* = 279)	10 CFU/0.6 mg	20 min	Reader	[41]
UCNPs—green core–shell disk	Spatial (3-line)	MMP-8; IL-1β; TNF-α	Gingival crevicular fluid	5.46/0.054/4.44 ng/mL	30 min	Reader	[42]
MNPs—MagQD NPs	Spectral + magnetic (2-color)	BoNT/A; SEB	Milk, juice	2.52; 2.86 pg/mL	30 min	Reader	[43]
MNPs—ZnCdSe/ZnS magnetic QD	Spatial (2-line)	Streptomycin; dihydrostreptomycin	Milk, tissues	0.08–1.78 μg/kg	25 min	Reader	[44]
MNPs—Janus Aushell-Fe_3_O_4_ (NIR)	Spatial (2-line, dual-mode)	H3N2; SARS-CoV-2 N	Respiratory swab	2; 7 pg/mL	30–40 min	Photothermal	[45]
MNPs—Polymer dot–MNP hybrids	Spatial (2-line, dual-mode)	AFB1; ZEN	Maize	2.15; 4.87 ng/mL	20 min	Reader	[46]
Au@Pdot nanohybrids	Spectral	CEA; CYFRA 21-1	Whole blood	0.12; 0.07 ng/mL	15 min	Colorimetric + Fluorescent reader	[47]
AuNR@Pdots	Spatial (2-line, dual-mode)	CAE; CA15-3	Buffer, Serum	0.096 ng/mL; 0.40 U/mL	15 min	Colorimetric + NIR-II fluorescence/photothermal	[48]
Urchin-Shaped Au–Ag@Pt	Spatial (2-line, multimodal)	*P. aeruginosa*, *S. aureus*, and *E. coli*	Bacterial spike	3 CFU/mL (*S. aureus*)	~2 h	Colorimetric + SERS + photothermal + catalytic	[49]
Platinum nanozyme (PtNZ)	Spatial (3-line)	AFP; hCG; CA125	Buffer, Serum	5.11 ng/mL; 1.55; 4.61 mIU/mL	35 min	Colorimetric	[50]
Coomassie bright blue R-250-labeled natural antibody network	Spatial (2-line)	CAP; Streptomycin	Milk	3; 20 ng/mL	11 min	Colorimetric	[51]
GO–Pt_30_–AuPt_5_ Nanozyme	Spatial (2-line)	SARS-CoV-2; H1N1	Respiratory swab	1.3; 8.4 pg/mL	18 min	Colorimetric	[52]

Multiplexing mode: spatial = spatially separated test lines, spectral = different reporter colors/Raman codes on a shared line, dual-mode = simultaneous colorimetric + spectroscopic readout, n.r. = not reported. † Reported as in compliance with regulatory thresholds rather than as a numerical LOD. LODs and assay times reported in this table are taken directly from the cited studies and are not intrinsic properties of the listed nanomaterials. They depend on the antibody affinity, the conjugate-to-antibody ratio, the strip geometry, and on any off-strip sample treatment such as pre-incubation, dilution, filtration or magnetic enrichment, and they are therefore not strictly comparable across rows.

**Table 2:** Common applications and limitations of nanomaterial reporters in multiplex LFIAs.

Reporter Family	Most Frequent Applications	Property Driving the Choice	Remaining Limitation
Multicolor QDs	Multi-mycotoxin and multi-residue food panels; CRP/PCT acute-care; COVID-19 IgM/IgG	Narrow, size-tunable emission enables 3–5-color spectral coding on one line	Cd toxicity; Cd-free alternatives still dimmer
SERS nanotags	Low-abundance clinical panels—cardiac, cytokine, Aβ42/Aβ40, respiratory viral antigens	Narrow Raman lines + 10^5^–10^9^ enhancement give ≥5 barcodes and sub-AuNP LODs	Needs portable Raman reader; hotspot reproducibility
UCNPs	Food and environmental panels in autofluorescent matrices; aquaculture multi-class; AIV subtyping	Anti-Stokes NIR excitation removes matrix background	Low quantum yield requires 980 nm laser; colloidal stability in serum/food
Surface-modified MNPs	Pathogens and biomarkers in complex matrices—whole blood, stool, and milk; oral-diagnostic panels	Magnetic pre-enrichment; dual-channel readout	Heavier/costlier reader; added sample-prep step
FNDs	Ultrasensitive single/duplex viral and serology assays	NV-centers photostability; ODMR lock-in-enabled background-free detection	ODMR readers are not yet widely available; few published multiplex works to date

**Table 3:** Recent AI-integrated LFIAs.

Reporter	ML Model	AI Function	Multiplexing	Target Analyte	Analytical Sensitivity (LOD)	Diagnostic Performance	Ref.
UCNPs	CNN	Classification	Single	Methamphetamine	below T/C floor (≈0.1 ng mL^−1^)	Acc 92%	[96]
UCNPs@SiO_2_ (MET/MOP-MAbs)	8 pretrained nets + transfer learning	Classification on IoT	Single	Methamphetamine Morphine	n.r.	Acc ≈ 99%	[95]
Calorimetric/SERS Rh@AgNPs	ANN and KNN	Deconvolution + classification	Single	Deoxynivalenol	4.21 pg/mL	Acc 98.8%	[93]
AuNPs	ResNet CNN and DyFormer	Classification	Single	Hepatitis B virus COVID-19	n.r.	Sens 95%/Spec 92%/Acc 94%	[99]
Commercial kit	Image processing algorithm	Quantification (no AI)	Single	Cryptococcal antigen	Surpasses visual reading	*p* < 0.0001 vs. visual	[100]
Polydopamine NPs	ViT and ResNet50 CNN	Classification + regression	Single	COVID-19 neutralizing antibody	160 ng/mL	n.r.	[91]
pCF-Apt-H1/H2 MNPs	SVM, RF, LR, and XGBoost	Classification + regression	Dual	EpCAM, Vim, and Colon CTCs	EpCAM 0.22; Vim 0.16 ng mL^−1^; CTCs 10 cells mL^−1^	Cancer-status acc 100%; pred acc 90.21%	[97]
Commercial kit	CNN (LeNet-5), SVM, k-NN, and RF	Classification	Single	SARS-CoV-2 N	n.r.	CNN 95.8%; RF 93.7%; SVM/k-NN < 83%	[90]
Commercial kit	Computer vision + regression	Quantification	Single	Influenza A and COVID-19	0.36–0.40 ng mL^−1^	Acc 95–96%	[101]
Commercial kit	CNN + transfer learning	Classification	Single	COVID-19 (antigens and antibodies)	n.r.	Sens 93–97%/Spec 96–99%/Acc > 95%	[92]
Commercial kit	YOLO, CNN, and LSTM	Temporal normalization + classification	Single	COVID-19, influenza A/B, Troponin I, and hCG	n.r.	Sens ≈ 96%/Spec 100%/Acc ≈ 97%	[102]
AuNPs	Signal processing + regression	Quantification	Single	Serum amyloid A protein	n.r.	Acc 94.23%	[103]
Au-Ag alloy SERS	MLR, MLP, and RF	Deconvolution + classification	Single	Interferon-γ (IFN-γ)	2.23 pg/mL	Acc 94.12%	[94]
AuNPs	CNN and Decision Tree	Classification	Multiplex (4)	SNP1, SNPs, SNP3, and SNP4	n.r.	Acc CNN 100%; DT 67–100%; Overall 97%	[104]
MNPs	SVM	Classification + regression	Single + multiplex (3)	Single: hCG Multiplex: cTnI, CKMB, and Myo	hCG: 0.014 mIU/mL cTnI/CKMB/Myo n.r.	n.r.	[98]

n.r. = Not reported; Acc = accuracy; Sens = sensitivity; Spec = specificity; MLR = multinomial logistic regression; MLP = multi-layer perceptron; RF = random forest; ViT = vision transformer. Diagnostic performance values follow the ‘sens/spec/acc (n)’ convention; where the original publication did not separate analytical from diagnostic metrics, the original reporting is preserved in the Performance column with an explicit n.r. flag.

**Table 4:** Challenges for implementation of AI in LFIA.

Challenge Category	Key Issue	Impact on Diagnosis	Possible Solutions & Strategies
Data Quality	Training on “clean” lab images only	Model failure when encountering real-world noise or poor-quality samples	Augmented training: noisy/blurred/skewed images; synthetic-data generation (e.g., GANs) to simulate rare error cases
Fluidic & Sample Variability	Patient-to-patient viscosity and “skewing”	Inaccurate quantification due to non-uniform flow or “fluidic lag”	Temporal normalization: LSTM analysis of wicking kinetics; ratiometric (T/C) analysis to correct for volume fluctuations
Hardware Disparity	Smartphone camera and sensor variability	Inconsistent results across different phone brands and models	On-strip color-calibration patches; transfer learning to fine-tune models for specific hardware profiles
Environmental Noise	Uncontrolled lighting and capture angles	Altered perceived intensity of bands leading to false readings	Preprocessing pipelines: Shadow removal, perspective correction, white-balance compensation
Regulatory Compliance	“Adaptive” AI algorithms that learn post-market	Difficulty in maintaining authorization as models evolve	Locked algorithm versions for clinical use; shadow-update validation; SaMD continuous monitoring frameworks
Clinical Trust	“Black box” nature of deep learning	Rejection of findings by medical professionals due to lack of transparency	Explainable AI (XAI): Grad-CAM heatmaps; per-result confidence scores; human-in-the-loop review for borderline cases

## Data Availability

No new data were created or analyzed in this study.
